# Antimicrobial activity against oral pathogens and immunomodulatory effects and toxicity of geopropolis produced by the stingless bee *Melipona fasciculata *Smith

**DOI:** 10.1186/1472-6882-11-108

**Published:** 2011-11-04

**Authors:** Silvana A Liberio, Antônio Luís A Pereira, Richard P Dutra, Aramys S Reis, Maria José AM Araújo, Nadia S Mattar, Lucilene A Silva, Maria Nilce S Ribeiro, Flávia Raquel F Nascimento, Rosane NM Guerra, Valério Monteiro-Neto

**Affiliations:** 1 Laboratório de Imunofisiologia, Universidade Federal do Maranhão, Av. dos Portugueses, SN, CEP: 65.080-040, São Luís, Maranhão, Brazil; 2 Departamento de Odontologia, Universidade Federal do Maranhão, Av. dos Portugueses, SN, CEP: 65.080-040, São Luís, Maranhão, Brazil; 3 Laboratório de Microbiologia, Universidade Federal do Maranhão, Av. dos Portugueses, SN, CEP: 65.080-040, São Luís, Maranhão, Brazil; 4 Laboratório de Farmacognosia, Universidade Federal do Maranhão, São Luís, Av. dos Portugueses, SN, CEP: 65.080-040, Maranhão, Brazil; 5 Laboratório de Microbiologia, Centro Universitário do Maranhão, Rua Josué Montello No. 1, CEP: 65.075-120, São Luís, Maranhão, Brazil

## Abstract

**Background:**

Native bees of the tribe Meliponini produce a distinct kind of propolis called geopropolis. Although many pharmacological activities of propolis have already been demonstrated, little is known about geopropolis, particularly regarding its antimicrobial activity against oral pathogens. The present study aimed at investigating the antimicrobial activity of *M. fasciculata *geopropolis against oral pathogens, its effects on *S. mutans *biofilms, and the chemical contents of the extracts. A gel prepared with a geopropolis extract was also analyzed for its activity on *S. mutans *and its immunotoxicological potential.

**Methods:**

Antimicrobial activities of three hydroalcoholic extracts (HAEs) of geopropolis, and hexane and chloroform fractions of one extract, were evaluated using the agar diffusion method and the broth dilution technique. Ethanol (70%, v/v) and chlorhexidine (0.12%, w/w) were used as negative and positive controls, respectively. Total phenol and flavonoid concentrations were assayed by spectrophotometry. Immunotoxicity was evaluated in mice by topical application in the oral cavity followed by quantification of biochemical and immunological parameters, and macro-microscopic analysis of animal organs.

**Results:**

Two extracts, HAE-2 and HAE-3, showed inhibition zones ranging from 9 to 13 mm in diameter for *S. mutans *and *C. albicans*, but presented no activity against *L*. *acidophilus*. The MBCs for HAE-2 and HAE-3 against *S. mutans *were 6.25 mg/mL and 12.5 mg/mL, respectively. HAE-2 was fractionated, and its chloroform fraction had an MBC of 14.57 mg/mL. HAE-2 also exhibited bactericidal effects on *S. mutans *biofilms after 3 h of treatment. Significant differences (p < 0.05) in total phenol and flavonoid concentrations were observed among the samples. Signs toxic effects were not observed after application of the geopropolis-based gel, but an increase in the production of IL-4 and IL-10, anti-inflammatory cytokines, was detected.

**Conclusions:**

In summary, geopropolis produced by *M. fasciculata *can exert antimicrobial action against *S. mutans *and *C. albicans*, with significant inhibitory activity against *S. mutans *biofilms. The extract with the highest flavonoid concentration, HAE-2, presented the highest antimicrobial activity. In addition, a geopropolis-based gel is not toxic in an animal model and displays anti-inflammatory effect.

## Background

Propolis is a generic name used for the product that results from the addition of the mandibular secretions of various bee species to resins these insects collect from the buds, flowers and exudates of different plants [1]. Distinct pharmacological activities of propolis have been demonstrated, including antimicrobial, anti-inflammatory, antitumor, cytotoxic, hepatoprotective and immunomodulatory properties, among others [2-4]. This diversity of pharmacological activities is related to quantitative and qualitative variations in the composition of different propolis samples [5,6].

Known pharmacological activities generally refer to propolis produced by *Apis mellifera*, the most common bee species in many countries and the main producer of honey in those countries [7]. However, some of these activities have also been observed in propolis produced by other bee species, including members of the tribe Meliponini [6,8]. Meliponines are stingless bees native to tropical and subtropical regions [9]. In the north and in some states of northeastern Brazil, *Melipona compressipes fasciculata *is the most important species for honey bee production and play an important role in flower pollination [10]. Stingless bees generally mix resinous material that they collect from plants with wax and soil and store large deposits of this material, called geopropolis, inside their hives. The final product is then used in a similar manner as propolis produced by *A. mellifera *[11].

In some countries, geopropolis has been used empirically by the population for wound healing, for the treatment of gastritis, and as an antibacterial agent [12]. Studies of ethanolic extracts of geopropolis produced by *M. compressipes *and *M. quadrifasciata anthidioides *have demonstrated its antimicrobial activity [13].

Although recent studies of *M*. *quadrifasciata *geopropolis have shown inhibitory activity against Gram-negative bacteria [14], this activity seems to be more pronounced against Gram-positive bacteria [6,8]. These results may be correlated with variations in chemical content, as has been observed for other types of propolis [13,15].

Due to its inhibitory effects on cariogenic microorganisms, such as *Streptococcus mutans *and *Lactobacillus *spp., and on bacterial enzymes (glucosyltransferases) involved in the cariogenic process, *A*. *mellifera *propolis has been proposed as an adjuvant for the control or prophylaxis of infectious diseases of the oral cavity, particularly dental caries [16-20].

Besides the pharmacological properties of propolis, its addition to different commercial products has drawn the attention of researchers for its possible toxicity, such as the triggering of hypersensitivity in the user. Most results show that the use of *Apis *propolis is relatively safe, since it presents low toxicity [21-25].

Thus, the objective of the present study was to investigate the antimicrobial activity of *M. fasciculata *geopropolis extracts against oral pathogens, its effects on the viability of *S. mutans *biofilms, and the chemical contents of the extracts. A gel prepared with a geopropolis extract was further analyzed for its antimicrobial activity on *S. mutans *and its immunotoxicological potential.

## Methods

### Preparation and fractionation of geopropolis extracts

Samples of geopropolis produced by *M. fasciculata *were collected from bee hives located in the municipalities of Palmeirândia and São Bento, a microregion of the Western Lowland of Maranhão (2°37'30"S 44°52'30"W), the ecosystems of which include mangrove swamps, floodplains, lakes, and babassu palm forests, and in the municipality of Fernando Falcão, a microregion of Alto Mearim and Grajaú (6°7'30"S 44°52'30"W), a savannah area. These municipalities are situated in the northwestern and central region of the State of Maranhão, in northeastern Brazil.

Individual geopropolis samples were weighed and mixed with ethanol (70%, v/v) to a final proportion of geopropolis/ethanol of 30/70 (w/v). Next, the samples were triturated in a homogenizer and macerated for 48 h. The samples were then filtered through filter paper to remove the inorganic portion (soil), concentrated in a rotary evaporator and air-dried at 40°C for 48 h. The three hydroalcoholic extracts obtained (HAE-1, HAE-2 and HAE-3) were stored in a refrigerator (4-8°C) until the time of analysis. The crude extract presenting the highest activity, HAE-2, was fractionated by liquid/liquid partitioning using solvents of different polarities (hexane and chloroform) [26]. The fractions obtained, HAE-2-HF (hexane fraction) and HAE-2-CF (chloroform fraction), were concentrated, dried and stored as described above.

### Preparation of a geopropolis-based gel

A geopropolis-based gel was prepared by mixing HAE-2 with 70% (w/v) Natrosol^® ^and 30% (v/v) propylenoglicol, as vehicle, for the formulation of a 5% (w/w) gel. Patent pending under No. PI0905583-5 INPI-RIMA, Brazil.

### Animals

C57Bl/6 mice, weighting 25-35 g, from the Central Animal Facility of the Federal University of Maranhão were used in the experiments with the geopropolis gel. The study was approved by the Ethics Committee for Animal Studies of the State University of Maranhão (No. 010/2007). All animals were cared for in accordance with the guidelines of the Brazilian Council of Animal Studies.

### *In vitro *evaluation of antimicrobial activity

Antimicrobial activity was evaluated using the agar diffusion method. For this study, 25 μL of extract was added to wells (5 mm in diameter) in agar plates as described previously [27]. The following culture media were used: brain-heart infusion agar (BHI agar, Difco) for *S. mutans*, Rogosa agar (Difco) for *Lactobacillus acidophilus*, and Sabouraud dextrose agar (Difco) for *Candida albicans*. Twenty-four-hour cultures of *S. mutans *(ATCC 25175), *L. acidophilus *(ATCC 4356) and *C. albicans *(ATCC 18804) were used. Cultures were adjusted with sterile saline to a density equivalent to the McFarland scale No. 0.5 (10^8 ^CFU/mL) and inoculated onto agar plates with a sterile swab. Ethanol (70%, v/v) and an aqueous solution of chlorhexidine gluconate (0.12%, w/w) were used as negative and positive controls, respectively. Plates of *S. mutans *and *L. acidophilus *were incubated at 37°C for 48 h in 5% CO_2. _*C. albicans *plates were incubated under aerobic conditions at 37°C for 24 h. After incubation, the diameters of the zones of inhibition were measured.

The minimum bactericidal concentrations (MBCs) were determined by the broth dilution technique using BHI broth (Difco). The final concentrations of tested hydroalcoholic extracts ranged from 12.5 to 0.19 mg/mL except the chloroform fraction (HAE-2-CF) for which concentrations ranged from 20 to 0.9 mg/mL. All assays were carried out in duplicate in three independent experiments. Dilutions of hydroalcoholic extracts were streaked on BHI agar plates and the MBC was determined. The MBC was defined as the lowest concentration of the extract that inhibited bacterial growth [28].

The minimum inhibitory concentration (MIC) for the geopropolis-based gel, against *S. mutans *(ATCC 25175), was determined by the broth dilution technique using BHI broth (Difco). The final concentrations of the product varied from 50 to 3.12 mg/mL and the minimum bactericidal concentration (MBC) was determined as described above.

### Inhibition of biofilm viability

The effect of HAE-2 on the viability of *S. mutans *(ATCC 25175) biofilms was evaluated as described previously [29,30], with some modifications. Briefly, for biofilm production, 24-well polystyrene cell-culture plates (TPP, Zellkultur und Labortechnologie, Switzerland) containing 1 mL tryptone-yeast extract broth supplemented with 1% sucrose were inoculated with approximately 1 × 10^6 ^CFU/mL of *S. mutans*. The culture medium was changed daily. On the fourth day, the culture supernatant was removed by aspiration, the bacterial biofilm was treated with HAE-2 diluted in 1% peptone broth at a concentration corresponding to four times its MBC (25 mg/mL), and bacterial counts were evaluated at different intervals. The negative control well contained only 1% peptone broth and chlorhexidine (0.12%) was used as the positive control [31]. The treatment solution was removed by aspiration at 1-h intervals for 4 h, and the biofilm was gently washed three times with phosphate-buffered saline (PBS), pH 7.2, scraped off, and suspended in 1 mL 1% peptone broth. The suspension was sonicated twice in a Model T7 sonicating water bath (Thornton, Brazil) at 50 W with three pulses of 10 s each at intervals of 5 s [30]. After homogenization, the suspension was diluted from 10^-1 ^to 10^-4 ^in PBS. Aliquots (100 μL of each dilution) were spread onto blood agar plates (blood agar base containing 5% sheep blood) and incubated in a 5% CO_2 _atmosphere at 37°C for 48 h. After incubation, CFU/mL were determined. These data were log_10 _transformed for analysis. All assays were carried out in quadruplicate.

A bactericidal effect was attributed to geopropolis extract when it resulted in a reduction of ≥ 3 log_10 _CFU/mL above baseline [30].

### Chemical characterization

Phenolic compounds, triterpenes and alkaloids were assayed in all extracts as described previously [32-34]. Total phenol concentration was assayed by spectrophotometry with the Folin-Ciocalteau reagent (Merck, Brasil) and 20% sodium carbonate solution for 2 h at room temperature in the dark. Known concentrations of gallic acid were used as standards. Measurements were made in a spectrophotometer (Cary 50 UV-VIS, Agilent, USA) at 760 nm and the results were expressed as a percentage of gallic acid [33]. The calibration equation for gallic acid (Sigma) was **y **= 0.06352**x **- 0.06132 (*R*^2 ^= 0.99) where **y **is absorbance and **x **is mg/mL of gallic acid.

The concentration of total flavonoids was determined by spectrophotometry in 2% aluminum chloride for 30 min at room temperature in the dark. Known concentrations of quercetin were used as standards and measurements were made at 425 nm. The results were expressed as a percentage of quercetin [33]. The calibration equation for quercetin (Merck) was **y **= 0.07347**x **- 0.00868 (*R*^2 ^= 0.99) where y is absorbance and **x **is mg/mL of quercetin. After linear regression analysis (CI 95%), both gallic acid and quercetin showed linear responses with different concentrations in the residue analysis. The relative standard deviations of the slopes were ≤ 5% for both standards (gallic acid, n = 9; quercetin, n = 6).

According to the Brazilian legislation, total phenols and flavonoids contents should be included in the chemical analysis as one of the quality criterion of propolis extracts [35].

The detection of triterpenes was performed by the Lieberman-Buchard reaction. Briefly, 2 mL of extract were mixed with 2 mL of chloroform. The mixture was filtered in the presence of Na_2_SO_4 _and 1 mL of acetic anhydride was added. After the addition of 3 drops of concentrated H_2_SO_4_, a change in color to brownish/red indicated the presence of triterpenes [32].

Total alkaloid content of extracts was determined using the Dragendorff's, Hager's, and Mayer's reagents [34]. Three aliquots of dried geopropolis extracts (10 mg) were dissolved in 1 mL of distilled water that had been acidified to pH 2.0-2.5 with 0.01 N HCl. Alkaloids were then investigated with 5 drops of each reagent separately in each tube.

### Animal experimental procedures

The animals were divided in three groups of 6 animals each, as follows: saline (S) - received a topical application of a sterile 0.85% NaCl (w/v) solution; GS - received a topical application of the pure gel base; and GP - received the gel base with HAE-2. The gel was applied topically to the oral cavity of the mice (25 μL/application), for a period of 1 minute, on four consecutive days.

### Assessment of biochemical parameters and cytokine production

For the biochemical and immunological analysis, blood samples were collected from the retro-orbital plexus on the seventh day after treatment. The samples were transferred to conical plastic tubes, with or without EDTA, and centrifuged at 1500 rpm for 10 minutes. Biochemical assessment consisted of a micro-assay to determine the serum concentrations of calcium, urea, cholesterol, triglycerides, albumin and glucose. The analysis were performed by means of automated procedures on an Architect - C8000 apparatus (Abbott^®^), using reagents from Labtest (Brazil). The concentrations of IFN-γ, IL-4, TNF-α and IL-10 were determined by immunoenzymatic assay (enzyme-linked immunosorbent assay; ELISA), according to the manufacturer's instructions (eBiosciences). Recombinant cytokines were used to generate a standard curve. The limit for detection in the assay was 2 pg/mL for IFN-γ, TNF-α, and IL-4, and 4 pg/mL for IL-10. The concentrations of cytokines were established using the coefficient of linear regression from values obtained on the standard curve.

### Macro- and microscopic assessment of the organs

The macroscopic analysis of the tongue, spleen, liver, stomach, and kidney considered the following parameters: size, weight, integrity and presence or not of changes visually detectable. Only the tongue was not weighed. For the histopathological analysis, all organs were fixed in 10% formol and embedded in paraffin. Next, 5 μm thick sections were cut on a microtome and stained with hematoxylin and eosin. The slides were examined in a light microscope with 20, 40 and 100× objectives. The following parameters were analyzed: the presence of vascular congestion, edema, inflammatory infiltrate, and hemorrhage. Tissue changes were scored as: 0 for absent, 1 for scarce, 2 for moderate and 3 for intense.

### Statistical analysis

The results were analyzed using SPSS for Windows 16.0 software (2007). Inhibition zones were compared using the Mann-Whitney test. Total phenol and flavonoid concentrations in the extracts as well as bactericidal effects of geopropolis on *S. mutans *biofilm viability were evaluated by analysis of variance (ANOVA) in conjunction with the Tukey multiple comparisons test. Data obtained with animals were expressed as mean ± standard deviation (X ± SD) for the immunological and biochemical results, and as mean ± standard error for the histopathological data. Statistical analysis consisted of analysis of variance (ANOVA), followed by Newman-Keuls test. Organ weights were subjected to Tukey's test and the histopathological data were analyzed by unpaired and single-tailed Student's *t*-tests. For all tests, effects were considered significant when p < 0.05.

## Results

Agar diffusion tests showed the antimicrobial action of geopropolis extracts (HAE-2 and HAE-3), with inhibition zones ranging from 10 to 13 mm for *S. mutans *and from 9 to 13 mm for *C. albicans*. No activity against *L. acidophilus *was detected (Table 1). HAE-2 and HAE-3 exhibited significant antimicrobial activity against *S. mutans *and *C. albicans *when compared to the negative control (p < 0.05). HAE-1 showed no antimicrobial activity against the microorganisms tested.

**Table 1:** *In vitro *activity of geopropolis extracts against *Streptococcus mutans*, *Lactobacilus acidophilus *and *Candida albicans*


**Extracts ^a^**	**Inhibition zone in mm ($\bar{X}$ ± SD)^b^**		
	
	***S. mutans***	***C. albicans***	***L. acidophilus***

**HAE-1**	0^d^	0^d^	0^d^
**HAE-2**	13 ± 1^cd^	13 ± 3^b^	0^d^
**HAE-3**	10 ± 2^cd^	9 ± 1^cd^	0^d^
**HAE-2-CF**	13 ± 2^cd^	0^d^	0^d^
**HAE-2-HF**	0^d^	0^d^	0^d^

**Chlorhexidine (0.12%)**	23 ± 1	15 ± 2	20 ± 3

**Ethanol (70%)**	0	0	0

^a ^Extracts of geopropolis from different municipalities, as shown in Table 1.

^b ^Average of agar diffusion tests performed in duplicate in three independent experiments.

^c ^Significantly different from negative control (70% ethanol).

^d ^Significantly different from positive control (0.12% chlorhexidine) (p < 0.05; Mann-Whitney test).

Evaluation of the antimicrobial activity of HAE-2 fractions revealed that only the chloroform fraction showed an inhibition zone when tested against *S. mutans *(13 mm). No antimicrobial activity of the hexane or chloroform fractions was observed against *C. albicans*. The MBC against *S. mutans *was 6.25 mg/mL for HAE-2, 12.5 mg/mL for HAE-3, and 14.5 mg/mL for the chloroform fraction HAE-2-CF. The MBC of geopropolis-based gel (prepared with HAE-2) against *S. mutans *was confirmed in 12.5 mg/mL.

**Figure 1:**
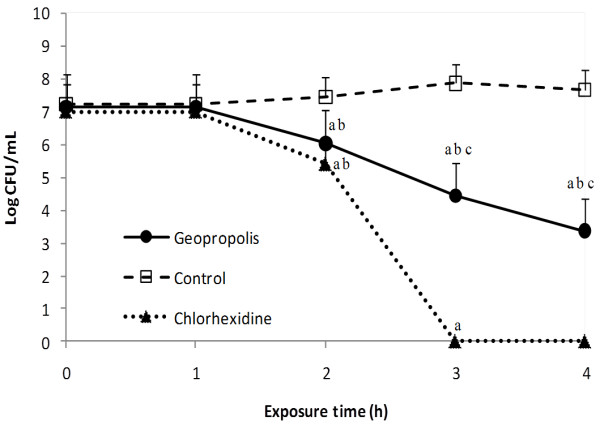
**Bactericidal effects of HAE-2 on the viability of *S. mutans *biofilms**. (a) p < 0.05 Effects in relation to time 0; (b) p < 0.05 Comparison of geopropolis activity on the biofilm viability in relation to the control (1% peptone broth) and (c) p < 0.05 Comparison of geopropolis activity on the biofilm viability relative to chlorhexidine. Comparisons were analyzed using the Tukey test.

Since HAE-2 presented a lower MBC than the other extracts and since the pathogenesis of the cariogenic process is related to the formation of a dental biofilm, we investigated whether this extract was able to inhibit the viability of *S*. *mutans *biofilms. A significant reduction in CFU/mL was observed after 2 h exposure of a biofilm to geopropolis extract HAE-2 or chlorhexidine (Figure 1). However, bactericidal effects were observed after 3 h treatment with geopropolis extract (reductions higher than 3log_10 _were obtained). Chlorhexidine treatment resulted in total elimination of *S. mutans *after 3 h exposure to the biofilm.

Chemical characterization of the geopropolis extracts indicated the presence of phenolic compounds in all samples. There were significant differences (p < 0.05) in total phenol and flavonoid concentrations among the samples analyzed (Table 2). Phenol content differed among the three extracts studied, with the lowest concentration being observed in HAE-2. In contrast, this extract contained the highest concentration of flavonoids. Triterpenes were detected in HAE-1 and HAE-2, but not HAE-3; alkaloids were not detected in any sample.

**Table 2:** Phenol and flavonoid concentrations of hydroalcoholic geopropolis extracts


**Extract**	**City**	**Total Phenol % (± SD) ^a, b^**	**Total Flavonoids % (± SD) ^a, c^**

**HAE-1**	Fernando Falcão	67.4 (2.0) ^d^	1.07 (0.04) ^f^
**HAE-2**	Palmeirândia	14.6 (2.3) ^e^	2.91 (0.22) ^d^
**HAE-3**	São Bento	51.2 (3.9) ^f^	1.11 (0.01) ^f^

^a ^Results are expressed as the average of assays carried out in triplicate.

^b ^Expressed as percentage of gallic acid.

^c ^Expressed as percentage of quercetin.

^d, e, f ^Values followed by different superscripts are significantly different from each other (p < 0.05; Tukey test).

**Figure 2:**
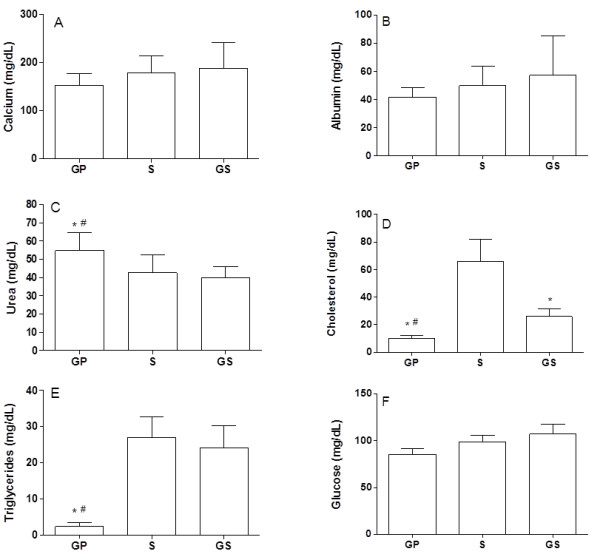
**Biochemical assessment**. Biochemical assessment of C57Bl/6 mice submitted to topical oral treatment with gel containing propolis (GP), compared to the group treated with the gel base (GS) or with saline (S). The serum concentrations of calcium (A); albumin (B); urea (C); cholesterol (D); triglycerides (E); and glucose (F) were determined. The results represent mean ± standard deviation of 6 animals/group. (*) p < 0.05 in relation to control and (#) p < 0.05 in relation to the other experimental group.

The experimental procedures with animals did not lead to any alteration in the serum concentration of calcium, albumin, and glucose in the GP group. On the other hand, animals in this group presented an increased concentration of urea and reduced concentrations of cholesterol, and triglycerides (Figure 2).

**Figure 3:**
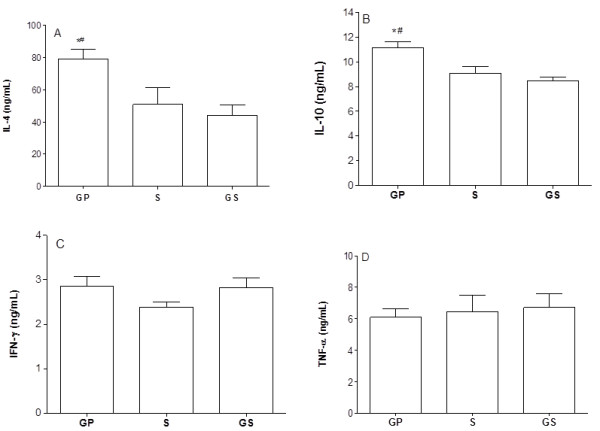
**Serum concentration of cytokines**. Serum concentration of cytokines in C57Bl/6 mice treated with topical oral gel containing propolis (GP) in comparison with the group treated only with the gel base (GS), or with saline (S). ELISA was used to determine the serum concentrations of IL-4 (A); IL-10 (B); IFN-γ (C) and TNF-α (D). The results represent mean ± standard deviation of 6 animals/group. (*) p < 0.05 in relation to the control and (#) p < 0.05 in relation to the other experimental group.

A significant increase in the production of IL-4 and IL-10 was observed in the GP group, while the production of IFN-γ and TNF-α was kept unaltered when compared to the S and the GS groups (Figure 3).

No significant changes were observed in the body weight of the animals and also in relation to the organs weight, with the exception of the stomach in the GS group (Table 3). Besides, no macro- or microscopic alteration was detected in the liver, kidney, or stomach. However, histopathological alterations, such as: vascular congestion, presence of edema, inflammatory infiltrate, and hemorrhage in the animal tongues, were significantly greater in the GS and S groups than in the GP group (Table 4).

**Table 3:** Effect of topical oral treatment with a gel based on geopropolis (GP) from *Melipona fasciculata *Smith on organ weight in mice.


**Groups**	**Final Weight (g)**	**Delta Weight**	**Organ weights (g)**			
			
			**Spleen**	**Kidney**	**Stomach**	**Liver**

**GP**	25 ± 4	0.2	0.08 ± 0.01	0.16 ± 0.03	0.42 ± 0.06	1.3 ± 0.2
**S**	28 ± 2	-0.2	0.10 ± 0.02	0.18 ± 0.02	0.33 ± 0.04	1.6 ± 0.2
**GS**	27 ± 2	-0.2	0.09 ± 0.01	0.17 ± 0.01	0.55 ± 0.08*	1.5 ± 0.2

* p < 0.05 (ANOVA, followed by Tukey's test.)

**Table 4:** Histopathological analysis of the tongue in mice that received topical oral treatment with a gel based on geopropolis (GP) from *Melipona fasciculata *Smith.


**Parameters Analyzed^a^**	**Treatment groups^b^**		
	
	**GP**	**S**	**GS**

**Vascular Congestion**	0.4 ± 0.1*^#^	1.8 ± 0.2 ^b^	1.6 ± 0.1
**Edema**	0 *^#^	1.2 ± 0.2	0.4 ± 0.1*
**Inflammatory Infiltrate**	0.2 ± 0.1*	1.2 ± 0.1	0 *
**Hemorrhage**	0.4 ± 0.1*	2.2 ± 0.2	0.4 ± 0.1*

(a) Tissue changes were scored as: 0 for absent, 1 for scarce, 2 for moderate and 3 for intense. Data represent the mean ± SEM of 6 animals per group

(b) GP - gel with geopropolis; GS - gel base and S - Saline

(*) p < 0.05 in relation to GP

(#) p < 0.05 in relation to S

## Discussion

In this study we observed that geopropolis can display inhibitory effects on some oral pathogens, without detectable toxicity in mice. Furthermore, an immunomodulatory activity, due to an increased anti-inflammatory cytokines, was also detected.

The inhibitory activity displayed by geopropolis against *S. mutans *and *C. albicans *was previously observed in extracts and fractions obtained from *Apis *propolis [15-17,19,20,36,37]. However, none of the three geopropolis extracts tested in this study were seen to have antimicrobial activity against *L. acidophilus*, unlike propolis extracts [38].

The inhibitory activity of geopropolis against *S. mutans *and *C. albicans*, but not against *L. acidophilus*, corresponds with the clinical significance of these microorganisms in oral cavity diseases. Both *S. mutans *and *L. acidophilus *ferment carbohydrates, forming organic acids that promote decalcification of dental enamel and dentine. Thus, the presence of these microorganisms is frequently associated with dental caries [39,40]. *S. mutans *is responsible for the initial demineralization of dental enamel, whereas bacteria of the genus *Lactobacillus *are related to lesion progression, i.e., after the initiation of the caries process [41]. Therefore, we speculate that a product containing *M. fasciculata *geopropolis, which mainly acts on *S. mutans*, may have important implications for caries-prevention strategies because *S. mutans *growth and biofilm formation are required for the onset of dental caries.

Little is known about the antibacterial and antifungal activities of geopropolis produced by native bees such as *M. fasciculata*, especially regarding its activities against oral microorganisms. A recent *in vivo *study showed that geopropolis produced by *M. fasciculata *promoted a reduction in salivary *S. mutans *counts [42]. However, the authors did not investigate the effects of geopropolis on other microorganisms, and no *in vitro *tests were performed. We found no other studies in the literature investigating the antimicrobial activity of geopropolis produced by *M. fasciculata *against *S. mutans*. Thus, the present findings are especially relevant since they underscore the potential for finding new active compounds that work against this microorganism in *M. fasciculata *geopropolis extracts.

The bactericidal activity of geopropolis extracts we observed against *S. mutans *biofilms is relevant not only because it confirms our *in vitro *results but also because it more closely reproduces the real conditions of the cariogenic process. Biofilm models are more relevant than studies which use planktonic cells because of the different growth of biofilm cells, their altered metabolism as a result of a high population density and their generally higher resistance to antimicrobial agents [43].

Despite the significant *in vitro *antimicrobial activity of chlorhexidine against *S. mutans *biofilms, its use in dentistry is controversial because of local side effects, which include discoloration of the teeth, tongue, restorations, and dentures; soreness of the oral mucosa; and irritation of taste buds [44].

*C. albicans *is considered a commensal microorganism, inhabiting the oral mucosa and other anatomic sites. However, in certain situations, this microorganism can cause opportunistic local or systemic infections that are often severe and difficult to control, especially in hosts with specific predisposing factors [45,46]. Thus, in addition to being used to control *S. mutans *growth, products formulated from *M. fasciculata *geopropolis might be used as adjuvants for the treatment of oral candidiasis and, ultimately, infections of other mucosa.

Chemical characterization of the extracts studied showed significant differences in the concentration of phenols and flavonoids among samples. The chemical composition of geopropolis produced by stingless bees in Brazil varies according to species. Moreover, available flora further influence the chemical composition of propolis [47]. According to Velikova et al. [6], members of the tribe Meliponini fly short distances and therefore use the first exudate sources that they encounter during their flights.

Only one of the propolis extracts (HAE-1) analyzed failed to show inhibitory activity against the microorganisms tested in this study, a finding that would suggest variations in the chemical composition of this sample. However, preliminary chemical analysis performed did not permit clear demonstration of such variation. Differences in its chemical content may have been influenced by its ecosystem because it originated in a savannah area, whereas HAE-2 and HAE-3 were prepared with geopropolis from an ecosystem whose vegetation comprised mangrove swamps, floodplains, lakes, and babassu palm forests.

Qualitative and quantitative differences in the composition of propolis have an important influence on its biological activity [48]. Among biologically active substances present, flavonoids are the main group contributing to the antimicrobial effects observed [21,49]. In this study, HAE-2 displayed the highest antimicrobial activity and had the greatest flavonoid concentration. Although the antimicrobial action of flavonoids is still controversial and conflicting results have been reported because of inter- and intra-assay variations in susceptibility tests, a link between flavonoids and inhibitory activity on microorganisms has been consistently demonstrated [50]. The antimicrobial activities observed here may be a product of high flavonoid concentration or, as reported for propolis produced by other bee species, a result of a synergistic action between flavonoids and other compounds present in these extracts [6,13].

Although some studies have reported cases of hypersensitivity reactions to *Apis *propolis [51,52], it is widely accepted that propolis does not present toxic effects[53,54], which confirms the results obtained with geopropolis in our study, since no macro and microscopic changes were detected in organs, which could indicate toxicity of the geopropolis-based gel on the mice.

Moreover, among the biochemical parameters studied, only an increase in the urea concentration was detected, but within the normal range for mice (41,97 mg/dl to 60,02 mg/dl), which does not indicate renal toxicity[55]. In fact, compounds like flavonoids, caffeic acid, and their esters present in propolis appear to prevent against membrane fragility in organs such liver and kidney, decreasing the level of urea and the leakage of liver enzymes into the circulation [54,56,57].

The reduction observed in the levels of cholesterol and triglycerides can be associated to the presence of antioxidants and flavonoids. Apparently, these compounds can act as inhibitors of lipid peroxidation (LPO) by scavenging polyunsaturated fatty acids' peroxy radicals and interrupt the chain reactions [58].

Despite not having shown signs of toxicity, the increased production of IL-4 and IL-10, cytokines associated with a Th2 response, suggests an anti-inflammatory activity for the product [59]. In fact, the histopathological assessment of the tongue corroborates these data, since tissue changes were significantly lower in the GP group, possibly because of the anti-inflammatory effects of the geopropolis. Tissue alterations in the other animal groups were probably resulting from their manipulation at the time of treatment and/or collection of the material.

The immunomodulatory effect displayed has been previously reported in investigations with *Apis *propolis [4,60-63]. However, we did not find any reports describing the effects of geopropolis from native bees on the production of cytokines, specifically IL-4 and IL-10.

The potential use of propolis (or geopropolis) in dentistry is promising not only due to its antimicrobial activity against oral pathogens, but also due to its other biological and pharmacological properties, which include anti-inflammatory, antitumor, antioxidant, hematostimulative, and immunomodulatory properties [2,4,64,65]. In addition, it is considered relatively non-toxic [21,22].

## Conclusions

In conclusion, our data indicate that geopropolis produced by *M. fasciculata *can exhibit antimicrobial activity against *S. mutans *and *C. albicans*. The activity against *S. mutans *was confirmed by further demonstrating the antibacterial effect of one extract on biofilm viability. The extract with the highest flavonoid concentration, HAE-2, displayed the highest antimicrobial activity. The gel maintained the antibacterial activity previously demonstrated in the geopropolis extract against *S. mutans*. Furthermore, no toxic effects were detected in mice treated with GP. On the other hand, an immunomodulatory action, due to the increase of anti-inflammatory cytokines, was observed. Our data demonstrate that geopropolis has the potential to be used for the control or prevention of diseases of the oral cavity, especially caries and candidiasis, as well as for treatment of inflammatory processes.

## List of Abbreviations

HAE: Hydroalcoholic extracts of geopropolis; MBC: Minimum bactericidal concentration; HAE-2-CF: Chloroform fraction of hydroalcoholic extract No. 2 of geopropolis; HAE-2-HF: Hexane fraction of hydroalcoholic extract No. 2 of geopropolis; BHI: Brain-Heart Infusion; ATCC: American Type Culture Collection; CFU: Colony-forming unit; PBS: Phosphate-buffered saline.

## Competing interests

The authors declare that they have no competing interests.

## Authors' contributions

SAL prepared the extracts, carried out the microbiological evaluation, organized the data analysis and drafted the manuscript. ALAP assisted with the data analysis and helped draft the manuscript. RPD carried out the chemical characterization and partitioned the extracts. ASR collected the geopropolis samples and helped with preparation of extracts. MJAMA helped with preparation of geopropolis extracts and animal experimental procedures. NSM helped with animal experimental procedures. LAS carried out histopathological analysis. MNSR participated in chemical characterization and helped draft the manuscript. FRFN helped with data analysis and manuscript revision. RNMG conceived the study, participated in its design and coordination, and critically reviewed the manuscript for important intellectual content. VMN conceived the study, coordinated the microbiological assays, and was involved in drafting the manuscript and revising it critically for intellectual content. All authors read and approved the manuscript.

## Pre-publication history

The pre-publication history for this paper can be accessed here:

http://www.biomedcentral.com/1472-6882/11/108/prepub
